# Heat-Stress Preconditioning Attenuates Behavioral Responses to Psychological Stress: The Role of HSP-70 in Modulating Stress Responses

**DOI:** 10.3390/ijms23084129

**Published:** 2022-04-08

**Authors:** Tal Belity, Michal Horowitz, Jay R. Hoffman, Yoram Epstein, Yaron Bruchim, Doron Todder, Hagit Cohen

**Affiliations:** 1 Department of Clinical Biochemistry and Pharmacology, Faculty of Health Sciences, Ben-Gurion University of the Negev, Beer-Sheva 8410501, Israel; belityt@post.bgu.ac.il; 2 Laboratory of Environmental Physiology, Faculty of Dental Medicine, The Hebrew University, Jerusalem 9112102, Israel; m.horowitz@mail.huji.ac.il (M.H.); yaron.bruchim@mail.huji.ac.il (Y.B.); 3 Department of Physical Therapy, Ariel University, Ariel 40700, Israel; jayho@ariel.ac.il; 4 Sackler Faculty of Medicine, Tel Aviv University, Tel Aviv and the Heller Institute of Medical Research, Sheba Medical Center, Ramat Gan 52621, Israel; yoram.epstein@sheba.health.gov.il; 5 Intensive Care, Veterinary Emergency and Specialist Center, Youth Village Ben Shemen, Ben-Shemen 7311200, Israel; 6 Beer-Sheva Mental Health Center, Ministry of Health, Anxiety and Stress Research Unit, Faculty of Health Sciences, Ben-Gurion University of the Negev, Beer-Sheva 8461144, Israel; doron.todder1@pbsh.health.gov.il

**Keywords:** hyperthermia, heat-stress, anxiety, heat shock protein-70, hypothalamus-pituitary-adrenal axis, preconditioning

## Abstract

Exposure to high ambient temperature is a stressor that influences both biological and behavioral functions and has been previously shown to have an extensive impact on brain structure and function. Physiological, cellular and behavioral responses to heat-stress (HS) (40–41 °C, 2 h) were evaluated in adult male *Sprague-Dawley* rats. The effect of HS exposure before predator-scent stress (PSS) exposure (i.e., HS preconditioning) was examined. Finally, a possible mechanism of HS-preconditioning to PSS was investigated. Immunohistochemical analyses of chosen cellular markers were performed in the hippocampus and in the hypothalamic paraventricular nucleus (PVN). Plasma corticosterone levels were evaluated, and the behavioral assessment included the elevated plus-maze (EPM) and the acoustic startle response (ASR) paradigms. Endogenous levels of heat shock protein (HSP)-70 were manipulated using an amino acid (L-glutamine) and a pharmacological agent (Doxazosin). A single exposure to an acute HS resulted in decreased body mass (BM), increased body temperature and increased corticosterone levels. Additionally, extensive cellular, but not behavioral changes were noted. HS-preconditioning provided behavioral resiliency to anxiety-like behavior associated with PSS, possibly through the induction of HSP-70. Targeting of HSP-70 is an attractive strategy for stress-related psychopathology treatment.

## 1. Introduction

Exposure to high ambient temperature is a stressor that influences both biological and behavioral functions. Heat-stress (HS) can result in physiological perturbations that include changes in cardiovascular and fluid balance [1,2]. These thermoregulatory disturbances stimulate the neuroendocrine system, important in the hypothalamic-pituitary-adrenal (HPA)-axis, in controlling and maintaining homeostasis [3,4].

The brain has a critical role in receiving sensory information and developing the physiological response to any stress, including thermal stress. Studies have shown that HS has an extensive impact on brain structure and function and may lead to neurological deficits, including neuronal loss [5,6,7]. Subsequently, these neurological changes have been shown to result in behavioral changes including increases in anxiety [8] and decreases in both learning and memory [6,9].

As the thermal stress is exacerbated, the risk of heat illness elevates [10]. Hyperthermia is associated with a systemic inflammatory response that can result in a multi-organ dysfunction and eventually heat-stroke, a life-threatening medical condition [10]. Following HS, an upregulation of pro-inflammatory mediators and markers of oxidative stress has been detected in the hippocampus of mice [11]. These markers included interleukin (IL)-1β, glial fibrillary acid protein, tumor necrosis factor α, nuclear factor-κB, cyclooxygenase (COX)-2 and induced-nitric oxide synthase. In contrast, to combat the increased inflammatory response to the elevation of core body temperature (Tb), upregulation in heat shock proteins (HSPs) within the central nervous system (CNS) has been noted, particularly the inducible 70 kDa (HSP-70) [12,13]. HSP-70 was upregulated in specific brain regions regulating the neuroendocrine response to a thermal stress including glial and vascular cells, hypothalamic nuclei including paraventricular, supraoptic and suprachiasmatic nuclei, the amygdala [12], hippocampal dentate gyrus (DG) and median eminence [13].

HSPs are a group of highly conserved proteins that function as molecular chaperones by facilitating the folding and refolding of proteins, mediating transmembrane transport of secretory proteins and targeting proteins for degradation [14,15]. Moran, Shapiro, Meiri, Laor and Horowitz [1,16] reported an improved cardiovascular response (lower heart rate and blood pressure) and lower rectal temperature in heat-acclimated rats, that could be explained, at least partially, by upregulation of HSP-70 during the acclimatation period [15,16,17]. Additionally, previous exposure to heat (HS-preconditioning) has been reported to stimulate an accumulation of HSP-70 in various tissue including the brain, which has been associated with an increase in heat tolerance [18,19]. For instance, elevations in HSP-70 were associated with lower cardiovascular and cerebral perturbations when mice previously exposed to a HS were subjected to a subsequent HS [18,19]. The underlying mechanism of heat-preconditioning is not entirely understood. Several investigations have suggested that previous exposure to a HS results in a shift to anti-apoptotic pathways [20], reduced oxidative stress and attenuated expression of pro-inflammatory cytokines, which are related to HSP-70 cytoprotective functions [15,21].

Heat acclimation as a thermoregulatory mediator has been previously described [15], but investigations exploring the role of HS-preconditioning in thermoregulation are lacking. Previous work has primarily focused on the physiological and cellular thermoregulatory implications of HS. The HPA-axis response [3,22], water/electrolyte balance impairments [3,23] as well as the immune response [11,22] to HS are well documented. The behavioral reaction to thermal insult, however, is limited [6,8,9,24]. Therefore, the first experiment of this investigation was to examine the effect of an acute HS (40 ± 1 °C, 2 h) on behavioral, physiological and cellular responses in rats. The level of several cellular markers of HS was evaluated in the dorsal hippocampus and hypothalamic PVN in response to HS. These markers included the glucocorticoid receptor (GR), HSP-70 and arginine vasopressin (AVP), which are three central players in stress responses and thermoregulation. Brain-derived neurotrophic factor (BDNF) and neuropeptide Y (NPY) are important for neuroplasticity and stress adaptation, as well as temperature regulation. Finally, given the close relationship between the neuroendocrine and the immune systems, COX-2 and ionized calcium binding adaptor molecule 1 (Iba-1) were evaluated. The second experiment of this investigation was to examine the effect of HS-preconditioning as a function of resiliency to psychological stress. In consideration that overexpression of HSP-70 has been previously demonstrated to result in an improved resiliency to a subsequent thermal stress [18,19], a third experiment was conducted. In this final experiment, levels of HSP-70 were manipulated using a pharmacological agent (Doxazosin) and an amino acid supplement (L-glutamine) [25] to examine whether increased levels of HSP-70 could account for the improved behavioral response to a psychological stress. 

## 2. Results

### 2.1. Experiment 1: The Effect of HS on Physiological, Cellular and Behavioral Responses

**Figure 1:**
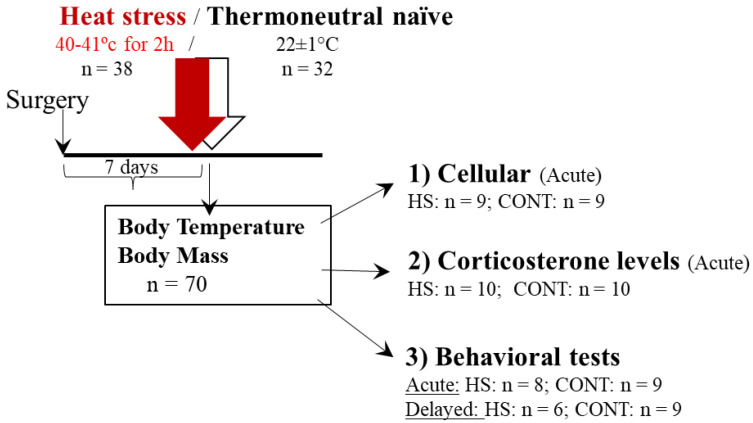
Study design–Experiment 1: The effect of heat-stress on physiological, cellular and behavioral responses. After a habituation period of seven days, animals were subjected to heat stress (HS) (40 ± 1 °C, 2 h). Control (thermoneutral, TN) animals were kept at room temperature (22 ± 1 °C) for the same period. Body mass and rectal temperature (Tre) were measured at baseline and every hour of the protocol. Core temperature was measured using telemetry throughout the entire protocol. After completion of the protocol, animals were sacrificed and their brains were collected for immunohistochemical analysis of chosen cellular markers. Trunk blood was used for circulating corticosterone measurement (corticosterone measurement). In a cohort of the animals, behavioral assessments were performed either immediately (acute response) or seven days after the HS exposure (delayed response) (Behavioral tests).

The experimental design used here is schematically depicted in Figure 1. 

#### 2.1.1. Physiological Measures

Three rats of HS group did not survive the protocol. 

**Figure 2:**
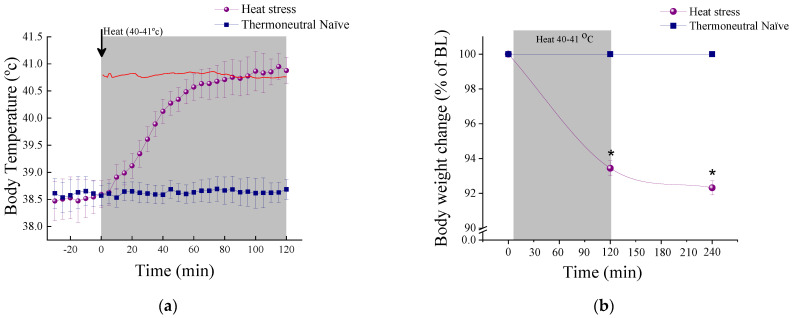
Physiological response to heat stress. (**a**) The dynamic change of core body temperature (Tb) over time during HS. (**b**) The change in body mass (BM) as percent change from baseline over time during HS. The red line represents the temperature inside the chamber along the experiment. Results displayed as mean ± S.E.M. * < 0.001.

Core body temperature: Figure 2a illustrates the dynamic change of Tb over time during HS and TN protocol. At baseline (−35 to 0 min) Tb did not differ significantly between the groups. RM-ANOVA showed an effect for groups (F(1,9) = 54.9, *p* < 0.0001), a time effect (F(23,207) = 51.2, *p* < 0.0001) and a group–time interaction effect (F(23,207) = 46.8, *p* < 0.0001). Tb was significantly increased in response to HS according to post hoc Bonferroni analysis.

Body mass: Figure 2b illustrates the change in BM as percent change from baseline. RM-ANOVA showed an effect for groups (F(1,25) = 654.7, *p* < 0.0001), a time effect (F(2,50) = 463.8, *p* < 0.0001) and a group–time interaction effect (F(2,50) = 447.3, *p* < 0.0001). 

A significant reduction in BM was noted in HS animals (*p* < 0.0001). After 2 h at room temperature of 22 ± 1 °C, additional reduction in BM was noted in HS animals (*p* < 0.004 compared to 2 h, *p* < 0.0001 compared to TN). 

Serum corticosterone concentrations: A significant increase in corticosterone concentrations was noted in response to HS (F(1,18) = 62.4, *p* < 0.0001) (HS: 704.5 ± 53.94 p gm/mL compared to TN: 217.5 ± 29.8 p gm/mL), indicative of increased HPA-axis activation in response to HS.

#### 2.1.2. Cellular Measures

**Figure 3:**
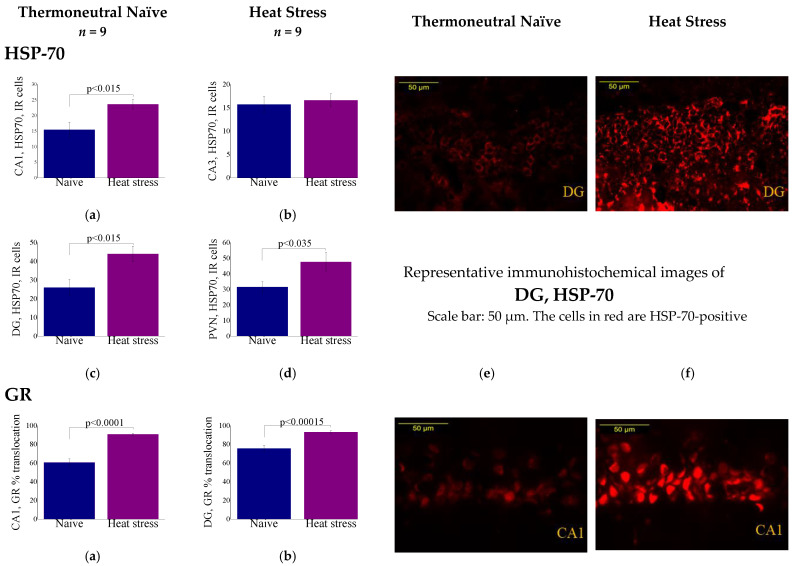

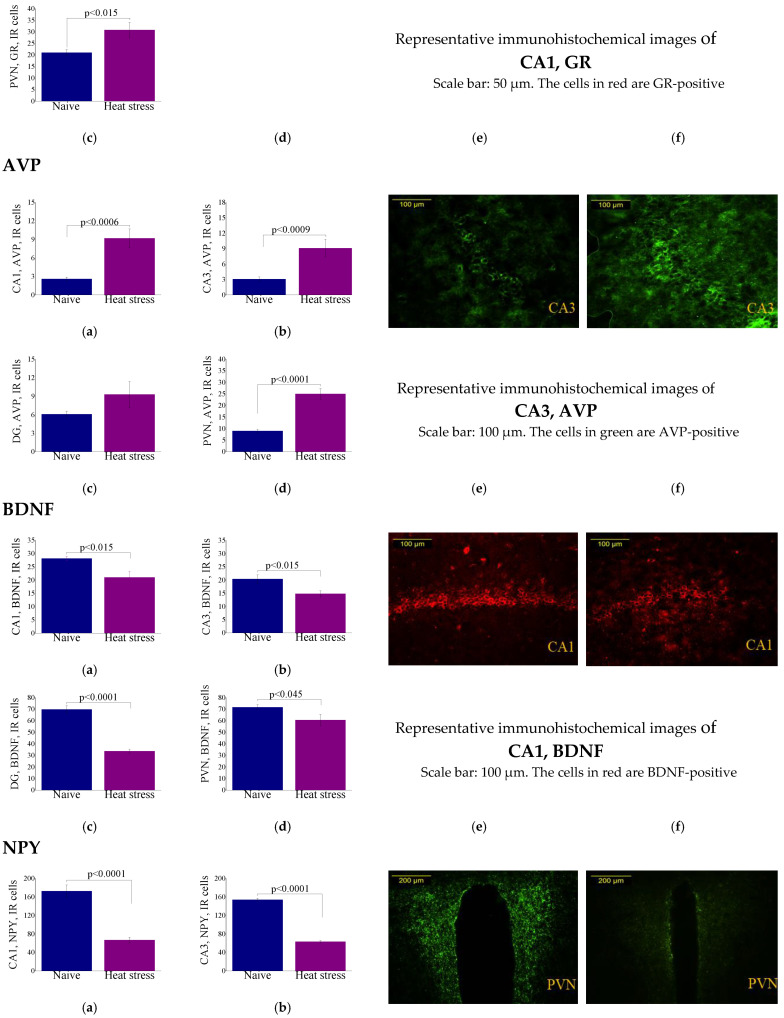

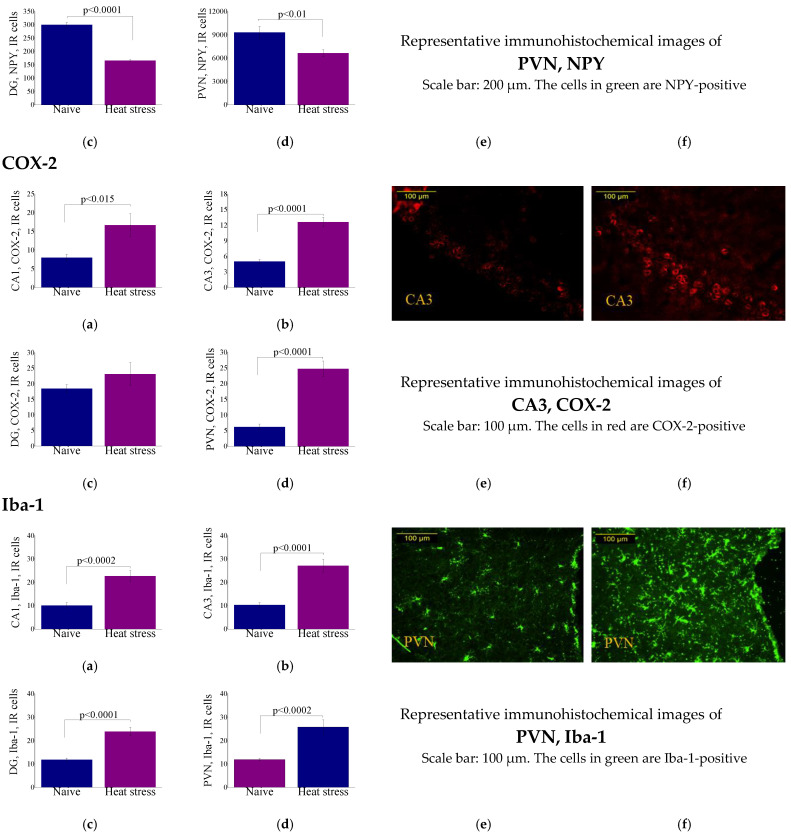
Cellular markers under basal thermoneutral naive and after heat stress. Immunoreactive-cell numbers and representative immunohistochemical images of HSP-70, GR, AVP, BDNF, NPY, COX-2 and Iba-1 positive cells within the CA1, CA3 and the DG of the hippocampus and the hypothalamic PVN (**a**–**e**). Results displayed as mean ± S.E.M.

Immunohistochemistry results of cellular markers under basal conditions (thermoneutral naive (TN)) and after heat stress (HS) are displayed in Figure 3. Immunoreactive (ir) cell numbers of HSP-70, GR, AVP, BDNF, NPY, COX-2 and Iba-1 positive cells within the Cornu Ammonis (CA) 1 and 3 and the DG of the hippocampus and the hypothalamic PVN were compared between the groups. Representative immunohistochemical images of each gene are displayed in Figure 3e,f.

HSP-70-ir cells: Significant differences in HSP-70-ir cell numbers were noted in the CA1 (F(1,16) = 7.7, *p* < 0.015, Figure 3a), DG (F(1,16) = 8.4, *p* < 0.015, Figure 3c) and PVN (F(1,16) = 5.6, *p* < 0.035, Figure 3d) subregions. No significant differences in HSP-70-ir cell numbers were noted in the CA3 subregion (Figure 3c). Bonferroni post-hoc analyses indicated that HSP-70-ir cell numbers in TN were significantly lower (*p* < 0.05) than in HS animals. Representative immunohistochemical images of HSP-70 within the DG of the hippocampus between basal thermoneutral naive (Figure 3e) and after heat stress (Figure 3f).

GR-ir cells: Significant differences in GR-nuclear localization were noted in the CA1 (F(1,16) = 55.3, *p* < 0.0001, Figure 3a), DG (F(1,16) = 24.9, *p* < 0.00015, Figure 3b) and PVN (F(1,16) = 7.7, *p* < 0.015, Figure 3c) subregions. Bonferroni post-hoc analyses indicated that nuclear localization of GR-ir cells in TN were significantly lower (*p* < 0.05) than in HS animals. Representative immunohistochemical images of GR within the CA1 of the hippocampus between basal thermoneutral naive (Figure 3e) and after heat stress (Figure 3f).

AVP-ir cells: Significant differences in AVP-ir cell numbers were noted in the CA1 (F(1,16) = 18.4, *p* < 0.0006, Figure 3a), CA3 (F(1,16) = 16.9, *p* < 0.0009, Figure 3b) and PVN (F(1,16) = 41.8 *p* < 0.0001, Figure 3d) subregions. No significant differences in AVP-ir cell numbers were noted in the DG subregion. Bonferroni post-hoc analyses indicated that AVP-ir cell numbers in TN were significantly lower (*p* < 0.05) than in HS animals. Representative immunohistochemical images of AVP within the CA3 of the hippocampus between basal thermoneutral naive (Figure 3e) and after heat stress (Figure 3f).

BDNF-ir cells: Significant differences in BDNF-ir cell numbers were noted in the CA1 (F(1,16) = 8.2, *p* < 0.015, Figure 3a), CA3 (F(1,16) = 8.1, *p* < 0.015, Figure 3b), DG (F(1,16) = 103.1, *p* < 0.0001, Figure 3c) and PVN (F(1,16) = 4.9, *p* < 0.045, Figure 3d) subregions. Bonferroni post-hoc analyses indicated that BDNF-ir cell numbers in TN were significantly higher (*p* < 0.05) than in HS animals. Representative immunohistochemical images of BDNF within the CA1 of the hippocampus between basal thermoneutral naive (Figure 3e) and after heat stress (Figure 3f).

NPY-ir cells: Significant differences in NPY-ir cell numbers were noted in the CA1 (F(1,16) = 51.4, *p* < 0.0001, Figure 3a), CA3 (F(1,16) = 531.7, *p* < 0.0001, Figure 3b), DG (F(1,16) = 216.3, *p* < 0.0001, Figure 3c) and PVN (F(1,16) = 8.6, *p* < 0.01, Figure 3d) subregions. Bonferroni post-hoc analyses indicated that NPY-ir cell numbers in TN were significantly higher (*p* < 0.05) than in HS animals. Representative immunohistochemical images of NPY within the PVN of the hypothalamus between basal thermoneutral naive (Figure 3e) and after heat stress (Figure 3f).

COX-2-ir cells: Significant differences in COX-2-ir cell numbers were noted in the CA1 (F(1,16) = 7.8, *p* < 0.015, Figure 3a), CA3 (F(1,16) = 64.8, *p* < 0.0001, Figure 3b) and PVN (F(1,16) = 49.4, *p* < 0.0001, Figure 3d) subregions. No significant differences in COX-2-ir cell numbers were noted in the DG subregion (Figure 3c). Bonferroni post-hoc analyses indicated that COX-2-ir cell numbers in TN were significantly lower (*p* < 0.05) than in HS animals. Representative immunohistochemical images of COX2 within the CA3 of the hippocampus between basal thermoneutral naive (Figure 3e) and after heat stress (Figure 3f).

Iba-1-ir cells: Significant differences in Iba-1-ir cell numbers were noted in the CA1 (F(1,16) = 24.1, *p* < 0.0002, Figure 3a), CA3 (F(1,16) = 35.0, *p* < 0.0001, Figure 3b), DG (F(1,16) = 40.5, *p* < 0.0001, Figure 3c) and PVN (F(1,16) = 23.8, *p* < 0.0002, Figure 3d) subregions. Bonferroni post-hoc analyses indicated that Iba-1-ir cell numbers in TN were significantly lower (*p* < 0.05) than in HS animals. Representative immunohistochemical images of Iba-1 within the PVN of the hypothalamus between basal thermoneutral naive (Figure 3e) and after heat stress (Figure 3f).

#### 2.1.3. Acute and Delayed Behavioral Response of HS

**Figure 4:**
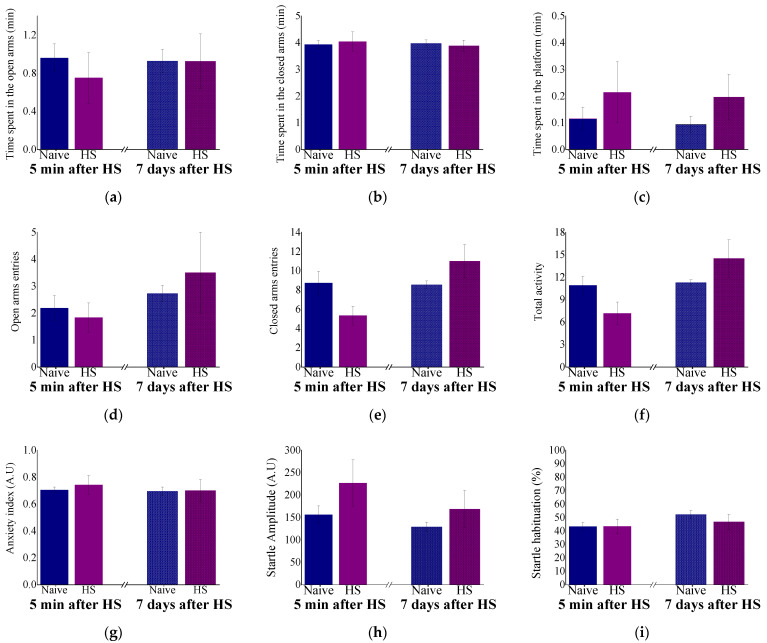
Acute and delayed behavioral response to heat stress. Acute and delayed behavioral response to HS. A.U = arbitrary units. Results displayed as mean ± S.E.M. No significant differences were noted in any of the elevated plus maze (EPM) (**a**–**g**) and the acoustic startle response (ASR) (**h**,**i**) parameters between the groups.

Acute and delayed behavioral responses are depicted in Figure 4. No significant differences were noted in any of the elevated plus maze (Figure 4a–g) and the acoustic startle response (Figure 4h,i) parameters between the groups.

### 2.2. Experiment 2: The Effect of HS-Preconditioning on Resiliency to Psychological Stress

**Figure 5:**
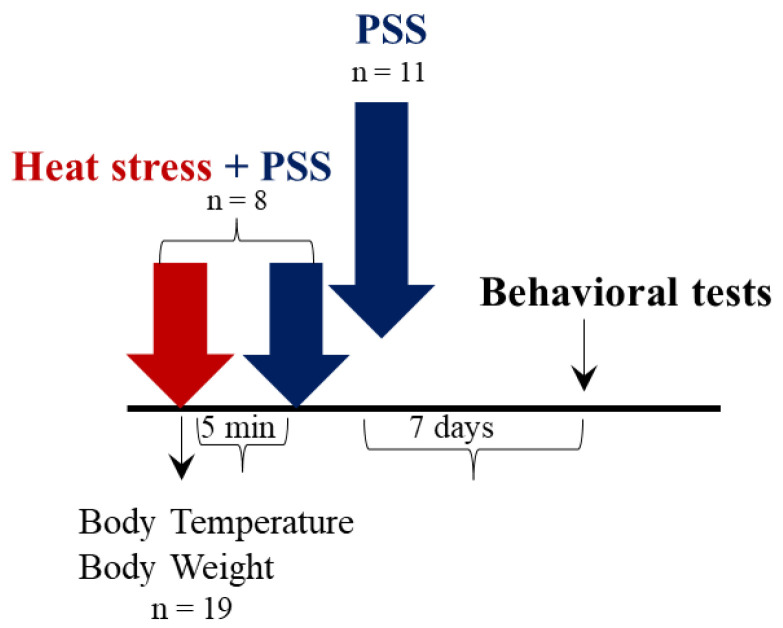
Study design–Experiment 2: The effect of HS-preconditioning on resiliency to psychological stress. After a habituation period of seven days, animals were subjected to HS and immediately were exposed to psychological stress, the predator-scent stress (PSS) (HS + PSS). Control animals were exposed to PSS only. Body mass and body temperature were measured at baseline, at every hour of the protocol and after 2 h at a room temperature (22 ± 1 °C). Behavior was assessed on day seven to examine the delayed behavioral response of HS-preconditioning to PSS.

The experimental design used here is schematically depicted in Figure 5. 

#### 2.2.1. Physiological Measures

A significant elevation in rectal temperature (Tre) was noted in HS + PSS animals (F(1,17) = 6.2, *p* < 0.02) compared to PSS animals (HS + PSS: delta Tre = 1.975 ± 0.16 °C vs. PSS: delta Tre = −0.7 ± 0.93 °C). In addition, a significant reduction (F(1,17) = 34.75, *p* < 0.0001) in BM was noted in HS + PSS animals compared to PSS animals (HS + PSS: % BM reduction = 5.019 vs. PSS: % BM reduction = 0).

#### 2.2.2. Behavioral Measures: The Effect of HS-Preconditioning on Resiliency to Psychological Stress

**Figure 6:**
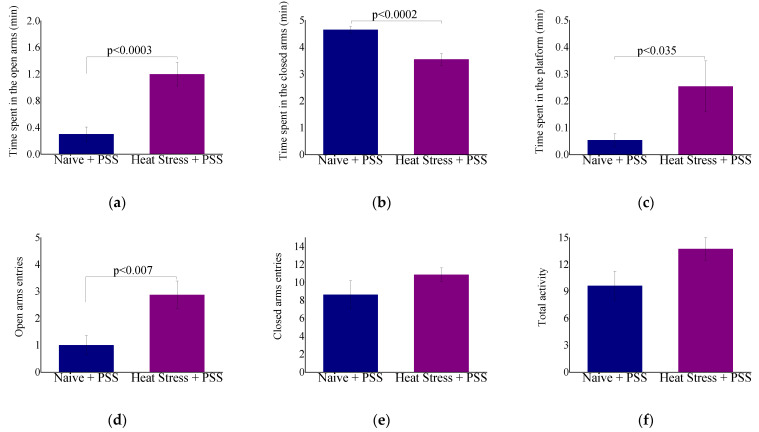

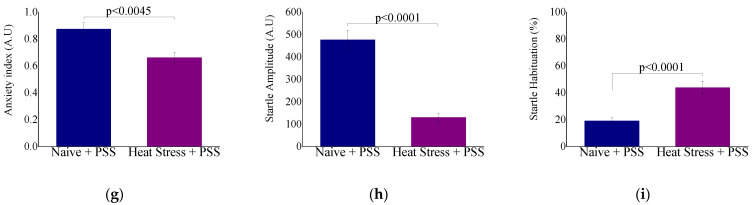
Behavioral response of HS-preconditioning to PSS (**a**–**i**). Behavioral response to HS-preconditioning to PSS. A.U = arbitrary units. Results displayed as mean ± S.E.M.

Behavioral responses to HS-preconditioning in the EPM and ASR paradigms can be observed in Figure 6. Time spent in the open arms (F(1,17) = 21.1, *p* < 0.0003, Figure 6a), time spent in the central platform (F(1,17) = 5.6, *p* < 0.035, Figure 6c), entries into open arms (F(1,17) = 9.6, *p* < 0.007, Figure 6d) and startle habituation (F(1,17) = 25.8, *p* < 0.0001, Figure 6i) were significantly higher in HS + PSS animals. In accordance, time spent in the closed arms (F(1,17) = 23.2, *p* < 0.002, Figure 6b), anxiety index (F(1,17) = 10.9, *p* < 0.0045, Figure 6g) and startle amplitude (F(1,17) = 44.6, *p* < 0.0001, Figure 6h) were significantly decreased in HS + PSS animals. No significant difference was noted in total exploration of the maze between groups (Figure 6f). These results suggest that HS-preconditioning might provide behavioral resiliency to the anxiety-like phenotype associated with PSS. 

### 2.3. Experiment 3: The Role of Heat-Shock Protein 70 in Modulating the Protective Behavioral Response to Predator-Scent Stress

**Figure 7:**
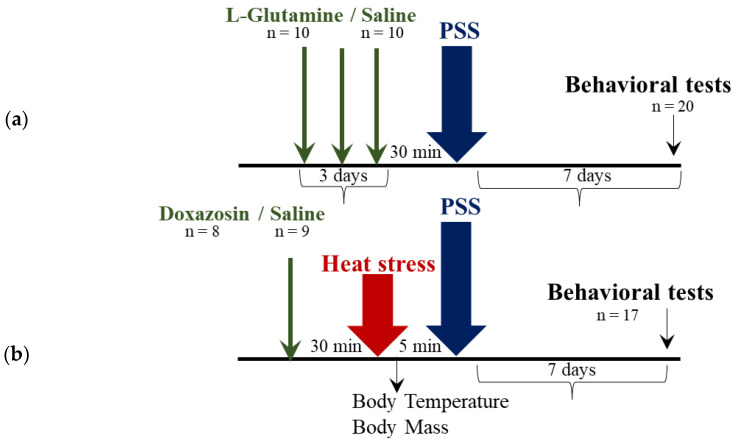
Study design–Experiment 3: The role of heat-shock protein (HSP)-70 in modulating the protective behavioral response to predator-scent stress (PSS). (**a**) L-glutamine: Animals were fed with the amino acid L-glutamine (0.375 g/kg) or vehicle via gavage for three days. On day three, animals were exposed to PSS and behavior was assessed on day seven. (**b**) Doxazosin: animals were injected with the α1-adrenoceptor antagonist Doxazosin (1.5 mg/kg i.p) or saline. Thirty minutes after the injection, animals were exposed to the HS and immediately after to the PSS. Body temperature and body mass were measured at baseline and every hour of the protocol. Behavior was assessed on day seven.

The experimental design used here is schematically depicted in Figure 7. 

#### 2.3.1. Physiological Measures

No significant difference was noted in Tre of animals that were treated with Doxazosin and saline (Doxazosin: delta Tre = 2.6 ± 0.2 ℃ and Saline: delta Tre = 2.26 ± 0.2 ℃). No significant difference was noted in BM of Doxazosin and saline animals (Doxazosin: % BM reduction = 6.01 and Saline: % BM reduction = 6.7).

#### 2.3.2. Behavioral Measures: Delayed Behavioral Response to Pharmacological Manipulation of HSP-70 Levels

**Figure 8:**
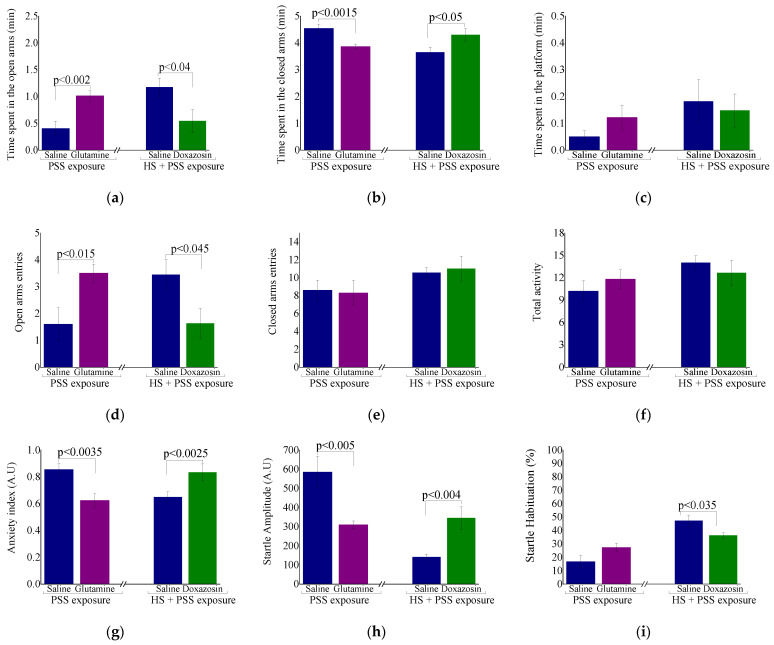
Behavioral response to manipulations of HSP-70 levels (**a**–**i**). Behavioral response to manipulations of HSP-70 levels. A.U = arbitrary units. Results displayed as mean ± S.E.M.

Behavioral responses in the EPM and ASR paradigms after HSP-70 manipulations are displayed in Figure 8. 

L-glutamine: Rats treated with L-glutamine and exposed to PSS spent significantly more time in the open arms (F(1,18) = 13.2, *p* < 0.002, Figure 8a), entered the open arms more often (F(1,18) = 7.2, *p* < 0.015; Figure 8d) and exhibited a significantly lower anxiety index (F(1,18) = 11.4, *p* < 0.0035, Figure 8g) than the vehicle-treated group. There was no difference in total activity in the EPM (Figure 8f). In the ASR, rats treated with L-glutamine and exposed to PSS showed a significantly lower mean startle amplitude than the vehicle group (F(1,18) = 10.65, *p* < 0.005, Figure 8h). No significant difference in startle habituation was found among the groups (F(1,18) = 3.7, *p* = 0.07, Figure 8i). 

Doxazosin: Injection of Doxazosin weakened the ability of HS to decrease the PSS-induced anxiety. In the EPM, rats treated with Doxazosin and exposed to HS + PSS spent significantly less time in the open arms (F(1,15) = 5.3, *p* < 0.04, Figure 8a), entered the open arms less often (F(1,15) = 5.0, *p* < 0.045; Figure 8d) and exhibited a significantly higher anxiety index (F(1,15) = 6.4, *p* < 0.0025, Figure 8g) than the saline-treated group. All rats showed similar overall activity in the EPM (Figure 8f). In the ASR, rats treated with Doxazosin and exposed to HS + PSS showed a significantly higher mean startle amplitude (F(1,15) = 11.8, *p* < 0.0045, Figure 8h) and a significantly lower startle habituation (F(1,15) = 5.5, *p* < 0.035, Figure 8i) than the saline-treated group. These findings suggest that the elevated levels of HSP-70 are involved in the anxiolytic effects of preconditioning HS before exposure to PSS.

## 3. Discussion

This study intended to examine the effect of HS on biological responses, with a specific emphasis on the behavioral response to a passive exposure to thermal stress. In addition, the behavioral response of HS-preconditioning to a psychological stress (PSS) was examined. Results of this investigation indicated that despite major physiological and cellular alterations, HS did not affect behavioral function. In addition, findings of this investigation suggested that exposure to an acute HS can diminish anxiety-like behavioral responses associated with PSS. As such, HS could serve as a preconditioning method to combat potential psychological stress.

### 3.1. The Effect of HS on Physiological, Cellular and Behavioral Responses

Acute exposure to HS resulted in a significant increase in Tre, a significant decrease in BM and a several fold increase in circulating corticosterone concentrations. This is consistent with previous findings indicating that corticosterone is sensitive to thermal stress in rodents [3,4,26,27].

### 3.2. Stress Associated Response: HS and Pyretic Osmoregulatory Genes

Immediately after HS, significant elevations of HSP-70-ir and GR-ir cells in the CA1, DG and PVN were noted. In CA1 and DG the upregulation of GR was accompanied by its translocation into the nucleus where GR could activate or repress target gene transcription. This is consistent with previous research examining HS applied to cell cultures [28,29] or whole body hyperthermia [30,31], which resulted in a loss of cytosolic GR binding capacity, a decreased amount of the GR protein present in the cytosolic fraction and its increase in the nuclei. 

Parallel responses of GR and HSP-70 suggest that both proteins are possibly inter-regulated or co-regulated by a common HS-activated transcription factor [32,33,34]. The HSP-70/GR ratio has been previously indicated to have possible implications on physiological response to stress [35]. The higher HSP-70/GR ratio observed in the hippocampus and the PVN suggested that HSP-70 was more available to influence GR transcriptional activity, which may include stabilizing DNA promoters [36], enhancing GR nuclear mobility [37] and GR nuclear recycling [38]. However, the association between HSP-70 and GR in relation to subcellular compartments distribution was not evaluated in this study.

In accordance with previous reports, HS resulted in a significantly higher number of AVP-ir cells in the CA1, CA3 and in the PVN [39,40]. It has been long recognized that AVP stimulates the secretion of adrenocorticotropic hormone from the pituitary gland and, therefore, is important for regulation of the HPA-axis during adaptation to stress [40,41,42]. In addition, AVP has an important role in maintaining water/electrolyte homeostasis and in thermoregulation [23,43]. However, accumulating evidence suggests that over-expression of AVP aggravated an existing brain injury [44]. For instance, treatment with a selective AVP receptor (V1a) antagonist after traumatic brain injury or stroke significantly diminished brain edema, blood-brain barrier disruption and neurological deficits [45,46,47,48]. Similar results were noted in AVP-deficient rats [49]. Thus, the activity of the vasopressinergic system in the current investigation could reflect a compensatory process or an unregulated, maladaptive, response to HS.

### 3.3. Neuroinflammation

Given the close relationship between the neuroendocrine and the immune systems, and as inflammation is suspected to mediate and exacerbate heat-related illnesses [11], the expression of COX-2-ir and Iba-1-ir cells was examined in response to HS. 

As expected, increased levels of both COX-2 and Iba-1 were noted in the CA1, CA3, DG and in the PVN after HS. This is in accordance with previous studies that reported on increased expression of pro-inflammatory markers, including COX-2, in mice’s hippocampus after thermal stress [11]. In response to pro-inflammatory stimulus, COX-2 converts arachidonic acid into prostaglandins, which, in turn, further stimulate the inflammatory response [50]. COX-2 activity in the CNS after brain injury has been described as deleterious. After global ischemia, an increased expression of COX-2 (mRNA and protein) was noted in the hippocampus of rats. Treatment with a COX-2 inhibitor (before, at 24 h and 48 h after the onset of ischemia) resulted in decreased expression of prostaglandins and an increased survival of CA1 neurons [51,52]. However, Baik, et al. [53] indicated that COX-2 inhibition aggravated kainic acid induced seizure, and increased neuronal death in the hippocampus of rats. Further, it has been suggested that COX-2-derived prostaglandins have protective properties in various tissues, including the nervous system [54,55,56]. Prostaglandins may have a role in regulation of the HPA-axis during stress by modulating the activity of neurotransmitters and neuropeptides [57]. Interestingly, Fujii, et al. [58] recently highlighted the importance of COX-2 in thermoregulation and noted that COX-2 contributed to the regulation of sweating during exercise in the heat in young men. Therefore, COX-2 activity in the CNS can be associated with both neurotoxic and neuroprotective processes. 

Microglia are considered as primary responders to adverse stimuli including stress. Emerging evidence suggests that these cells participate in neuronal functions including neuronal activity and synaptic plasticity during both homeostatic and pathological states [59]. In addition to being the main source of pro-inflammatory cytokines, microglia are involved in tissue repair and wound healing processes in the CNS, aimed at maintaining brain health [59,60]. The results of this study suggest that early activation of local microglia following an acute HS is beneficial and can provoke a variety of adaptive effector functions, such as phagocytosis of cellular debris and expression of neurotrophins, glutamate transporters and HSPs, which help mount the appropriate (adaptive) physiological and behavioral responses following thermal stress [59]. 

### 3.4. Neuroplasticity

Neurotrophies, in particular BDNF, regulate neuronal survival, differentiation and growth, and modulate many aspects of neuronal plasticity [61,62,63,64,65]. Converging evidence implicates the crucial impact of neurotrophins to CNS function in health and disease [62]. In the current study, HS significantly decreased the number of BDNF-ir cells. The effect of an acute HS on BDNF expression in different brain areas has yet been investigated, but there have been some inconsistent reports on BDNF expression in response to different acute stresses. In rats that were subjected to an acute stress (immobilization or forced swim), the expression of BDNF was either decreased [66], increased [67] or remained intact [68] in the hippocampus, but increased in the hypothalamic PVN [69]. While chronic alternations of BDNF expression (most commonly downregulation) are associated with psychopathologies, including depression and anxiety (for review, [70]), the response of BDNF to an acute stress remains elusive, and so is its role in thermoregulation. Recently, it has been suggested that BDNF and glucocorticoids (GCs) share homeostatic effects in the CNS [70,71]. Increased GCs in response to either stressful insult or GCs treatment, attenuated BDNF levels via direct binding of GR to regulatory sequence on the *bdnf* gene promotor [62]. High levels of BDNF, however, induced corticotropin-releasing hormone release through activation of the GR signaling pathway [72]. Taken together, it is reasonable to assume that the decreased BDNF levels observed after HS are the result of increased corticosterone concentrations, representing a normal stress response aimed at restoring physiological homeostasis. 

NPY is expressed mainly in neurons of the brainstem, hypothalamus and limbic system and numerous reports have implicated its importance in stress-adaptation processes as an anxiolytic agent (for review, [73]). In support of this, central administration of NPY attenuated corticosterone and epinephrine expression in acutely heat-exposed fasted chicks [74]. Additionally, the NPYergic system has been associated with behavioral resilience to psychological stress (PSS) in rats [75]. A decrease in rectal temperature along with increment in HSP-70 expression were noted in chicks that were exposed to HS and centrally administrated with NPY, indicating a thermoregulatory role of NPY during thermal stress [76]. Unlike the results obtained in this study, NPY mRNA expression was significantly increased in the hypothalamus of chicks [77] and rats [78] in response to an acute HS or restraint stress, respectively. The reason for this discrepancy is unclear but could be related to differences in the type of stressor, exposure duration and timing of outcomes measurement. Reciprocal relation exists between BDNF and NPY signaling pathways. Previous investigation noted that overexpression of the NPY neuron has led to a parallel upregulation of BDNF mRNA [79]. Others have shown that BDNF is a regulator of the functional and morphological expression of the NPY neuron [80]. Therefore, increased corticosterone concentrations and decreased BDNF levels have led to a decrease in NPY levels in the acute phase response to HS.

### 3.5. HS-Mediated Behavioral Resiliency

Extensive cellular changes were detected in the hippocampus and the hypothalamic PVN after a single exposure to HS. These selected genes regulate protein trafficking, translation and gene transcription, and enable changes to functional and structural plasticity, changes that could be interpreted either as an unregulated/maladaptive response to thermal damage to CNS homeostatic sites, or as an adaptive thermoregulatory response. The pattern of change in requisite molecular signaling genes was previously found to correspond with the structural and behavioral responses to the *maladaptive* phenotype to stress [75,81,82,83,84], signify a potentially more vulnerable state physiologically. The preceding increase in body temperature may increase feelings of fatigue or physical exhaustion and therefore to lower exploration (activity) and behavioral perturbations. However, in contrast to previous investigations [6,8,9,24], in this study HS did not affect either the acute or the delayed behavioral response). Importantly, in these previous investigations heterogenous methodology was reported including the HS protocol (ambient and internal temperature, duration and number of HS exposures) and timing of behavioral assessment. Taken together, our results indicate that HS-induced central biochemical (cellular) and physiological modifications that, in turn, resulted in an anxiolytic behavioral response.

### 3.6. HS Induced Preconditioning via Cross Tolerance Mechanism

The behavioral effect of HS-preconditioning to PSS was examined. Current investigations aimed at understanding the effectiveness of preconditioning mainly consisted of animal models of ischemic-preconditioning to combat physical injury such as surgical procedures, while the use of this method to diminish psychological stress-related deficits has not been examined. It is worth noting that preconditioning has been found to be protective to various tissues such as the kidney, liver, heart and the brain, but only in animal models [85,86]. Here, we present evidence that a single exposure to HS may, in some cases, diminish detrimental effects of psychological stress. The persistent anxiety-like behavioral response associated with PSS do not appear to exist when animals are exposed to thermal stress immediately prior the PSS.

### 3.7. Preconditioning and HSP-70 Levels

Cellular dynamics triggered by HS are of great importance for implementation of protective effects. For example, different organs including the heart and brain react to thermal stress by activating a complex cascade of cellular events leading to a shift towards a defensive (preconditioned) phenotype characterized by upregulation of HSPs [87,88,89]. HSPs enhance cell capacity to handle denatured proteins, prevent aggregation and facilitate the renaturation or degradation of these proteins [15], thus making the organism more tolerant to additional stress.

Increased levels of HSP-70 after HS may have improved GR-heterocomplex activity and facilitated the transcription (or repression) of relevant genes that attenuated the anxiety-like behavior associated with PSS. To test this hypothesis, in the final experiment of this investigation, animals were fed with the amino-acid L-glutamine for three days before the exposure to PSS. Oral administration of L-glutamine has been previously indicated as an inducer of HSP-70 levels in rats [25]; therefore, the animals were not also exposed to HS. As expected, animals treated with L-glutamine exhibited behavioral resiliency to the PSS according to the EPM and the ASR paradigms. In accordance, a single injection of Doxazosin 30-min before the exposure to both HS and PSS increased the animals’ susceptibility to develop the anxiogenic phenotype in response to PSS. Doxazosin is a potential attenuator of HSP-70 levels [90,91]. Similarly, Zhu, et al. [92] previously found that hyperthermia inhibited the development of anxiety-like behaviors underlying chronic unpredictable stress in mice, and suggested that the protective role of hyperthermia is associated with an up-regulation of HSP-72 in the hippocampus [92]. 

The hypothalamic GR is a main mediator of feedback inhibition of the HPA-axis, therefore its increased levels, in addition to enhanced availability of its chaperon HSP-70 and increased expression of AVP could constitute at least one of the mechanisms of HPA-axis activity in HS animals subjected to subsequent acute (psychological) stress. A proper/adequate activation of the HPA-axis in response to stressogenic experiences, either as part of the normal stress response or because of a high dose of replacement, would be adaptive. The proper activation may not only result in a better preparation and response to the acute demands of physical and emotional stressors (fight or flight) but may also result in a faster recovery with the passage of the threat [93]. 

Our study comes with several limitations which should be addressed in future investigations. First of which is the relatively small sample size, although the overall sample size was sufficient for statistical analyses. In addition, a continuous core body temperature measure as well as a complete cellular analysis in experiments 2 and 3 would have provided a better understanding of the heat strain during HS and possibly also of the mechanism(s) underlying HS preconditioning. Finally, L-glutamine and Doxazosin were not established as mediators of specific central HSP-70 levels. While L-glutamine administration resulted in an elevation of HSP-70 in several tissues including the brain of rats, it is possible that other proteins were affected and were responsible for the adaptative phenotype seen after HS. In addition, the effect of Doxazosin administration on HSP-70 levels was not tested and was chosen as a pharmacological agent for this investigation based on a previous observation indicating a similar effect of other α-adrenergic antagonists. 

Taken together, short term (2 h) exposure to HS (40–41 °C) led to extensive physiological and cellular changes but did not induce any behavioral response. These changes could be interpreted either as an unregulated event due to thermal damage to central nervous system homeostatic sites, or as an adaptive thermoregulatory survival mechanism. HS-preconditioning to PSS attenuated behavioral responses of psychological stress, possibly through the induction of HSP-70. We hypothesize that in response to stress strategies, increasing intracellular HSP-70 might be a worthwhile approach to treat stress-related sequela. After stress exposure pharmacological induction of HSP-70 can initiate (and maintain) the behavioral stress responses by modulating neurohormonal and inflammatory pathways, as well as impeding apoptosis processes via direct and indirect means, enabling the animals to better prepare for, respond to, and cope with the acute demands of physical and emotional threats to re-establish homeostasis. Therefore, targeting of HSP-70 is an attractive strategy for stress-related psychopathology treatment. 

## 4. Materials and Methods

All procedures were performed under strict compliance with ethical principles and guidelines of the National Institutes of Health (NIH) Guide for the Care and Use of Laboratory Animals. All treatment and testing procedures were approved by the Animal Care Committee of Ben-Gurion University of the Negev, Israel (IL-91-11-2019(D)) and by the Ethics Committee for Animal Experimentation of The Hebrew University, Jerusalem, Israel (IL-26-06-2017).

### 4.1. Animals

One hundred and twenty-six adult male *Sprague-Dawley* rats (Envigo RMS Israel Ltd.) weighing 200–250 g were habituated to housing conditions for a minimum of seven days. Animals were housed two per cage in a vivarium with stable temperature of 22 ± 1 °C and a 12 h light/dark cycle (lights on: 8:00 h) with unlimited access to food and water. BM was monitored biweekly.

### 4.2. Experimental Design

Three separate groups of animals were used in three different experiments designed to test physiological, cellular and behavioral responses to HS, the behavioral effect of heat-preconditioning to psychological stress and to test a possible mechanism of HS-preconditioning to psychological stress. Figure 1, 5, 7 display the study design.

Experiment 1 (*n* = 70): The effect of HS on physiological, cellular and behavioral responses. Rats were randomly assigned into an HS (*n* = 38) or thermoneutral (TN) (*n* = 32) group. The animals in HS were subjected to a passive heat stress (40 ± 1 °C) for 2 h in a thermal chamber, while animals in TN were held at room temperature (22 ± 1 °C) for 2 h. Physiological measures included rectal temperature and BM that were obtained at baseline and every hour of the protocol. Core body temperature was measured using telemetry in a small cohort of the animals (HS: *n* = 7; TN: *n* = 7). After completion of the protocol, animals were sacrificed and their brains were collected for immunohistochemical analysis of chosen genes. Trunk blood was used for circulating corticosterone measurement. In a cohort of the animals (*n* = 32), behavioral assessments were performed either immediately after the HS (acute response) or seven days after HS exposure (delayed response). Behavioral measures included the elevated plus maze (EPM) and the acoustic startle response (ASR) paradigms. 

Experiment 2 (*n* = 19): To determine the effect of HS preconditioning on resiliency to psychological stress, animals were subjected to the same thermal stress protocol as detailed above and then were exposed to the predator scent stress. The PSS is an animal model that has been previously established as a valid and effective method of examining biomolecular and physiological parameters of specific response patterns of stress [94,95]. Animals were randomly assigned to either a HS + PSS (*n* = 8) or a PSS only (*n* = 11) group. HS + PSS animals were subjected to HS and immediately were exposed to PSS. PSS animals served as control and were exposed to PSS without HS. Tre and BM were measured at baseline, every hour of the protocol and after 2 h of recovery at room temperature (22 ± 1 °C). Behavior was assessed for the two groups on day seven in the EPM and ASR paradigms to examine the delayed behavioral response of HS-preconditioning to PSS. 

Experiment 3 (*n* = 37): To examine the role of HSP-70 in modulating the protective behavioral response to PSS, animals were randomly assigned into one of two groups: 

L-glutamine: Animals were fed with the amino acid L-glutamine (0.375 g/kg) (*n* = 10) or vehicle (*n* = 10) via gavage for three days. Oral administration of L-glutamine was previously found to increase HSP-70 levels in the lung and gut of heat-stressed rats [25]. On day three the animals were exposed to PSS (15 min) and behavior was assessed on day seven in the EPM and ASR paradigms.

Doxazosin: Animals were injected with the α1-adrenoceptor antagonist Doxazosin (1.5 mg/kg i.p (Dexcel Pharma, Israel) (*n* = 8) or saline (*n* = 9). The administration of a α1-adrenoceptor antagonist (prazosin) before stress exposure attenuated the expression of HSPs, including HSP-70, in several tissues in rats [90,91]. Doxazosin is a long acting α1 adrenergic antagonist (half lifetime 22 h), therefore is a suitable option for this experiment in which we investigated delayed/long-term behavioral effect. 30 min after the injection of Doxazosin animals were exposed to HS and PSS as detailed above. Behavior was assessed on day seven in the EPM and ASR paradigms. Doses of l-glutamine and Doxazosin were chosen based on previous studies [25,90,91].

### 4.3. Surgical Procedure

Rats were peritoneally implanted with radio transmitters (DST nano-T N1983, Mercury) in a surgical procedure by a trained veterinary surgeon under sterile conditions (*n* = 14). Rats were anaesthetized with a combination of ketamine (70 mg/kg) and xylazine (6 mg/kg) i.p. Ketoprofen (5.0 mg/kg) was provided for postsurgical analgesia. Seven days after the surgery, rats were exposed to HS or neutral protocols and were immediately sacrificed for immunoreactivity analysis of specific brain regions. Probes were programmed to log core temperature at 5-min intervals and were removed posthumously.

### 4.4. Rectal Temperature (Tre) and Body Mass (BM) 

Rectal temperature and body mass were measured at baseline and every hour of the protocol. Tre was measured with a lubricated digital thermometer probe (YL402 thermistor) by hand and BM was obtained using a top-loading balance accurate to ±0.1 gr.

### 4.5. HS Protocol

Conscious rats were exposed to a well-established protocol of passive HS previously described [2,96]. HS was conducted for 2 h in a climatic chamber at a controlled temperature of 40 ± 1 °C and relative humidity of 30–35%. To avoid handling stress, HS rats were transferred in their home cages into the climatic chamber. TN rats stayed in their home cage under normal conditions without being handled. Food and water were removed during the entire study for both groups.

### 4.6. Immunohistochemistry

Tissue preparation: 3–5 min after completion of the protocol, animals were deeply anesthetized (ketamine and xylazine mixture) and perfused transcardially with cold 0.9% physiological saline followed by 4% paraformaldehyde (Sigma-Aldrich, Rehovot, Israel) in 0.1 M phosphate buffer (pH 7.4). Brains were quickly removed, post-fixed in the same fixative for 12 h at 4 °C, and cryoprotected overnight in 30% sucrose in 0.1 M phosphate buffer at 4 °C. Brains were frozen on dry ice and stored at −80 °C. Serial coronal sections (10 µm) at the level of dorsal hippocampus were collected from each animal using a cryostat (Leica CM 1850, Germany) and mounted on coated slides.

Staining: Sliced sections were air dried and incubated in frozen ethanol (4 min). After washes in phosphate-buffered saline (PBS) (Sigma-Aldrich, Rehovot, Israel), the sections were incubated for 120 min in a blocking solution: 6% bovine serum albumin (BSA) and 5% serum in PBS, and then incubated overnight at 4 °C with primary antibodies against NPY mouse monoclonal anti-NPY antiserum (1:400), (product code: sc-133080, Santa Cruz Biotechnology, Inc., Heidelberg, Germany), BDNF (rabbit polyclonal anti-BDNF antiserum (1:200), product code: sc-ANT-010, Alomone Labs, Jerusalem, Israel), anti-COX-2 (mouse monoclonal anti-COX-2 antiserum (1:250), product code: sc-166475, Santa Cruz Biotechnology, Heidelberg Germany), anti- HSP-70 (rabbit polyclonal anti-HSP-70 (1:250), product code: SPC-103, Stress-Marq Biosciences) and anti-AVP (rabbit polyclonal anti-AVP antiserum (1:100), product code: ab68669, Abcam). After three washes in PBS, sections were incubated in DyLight-488 labeled goat-anti-rabbit IgG (NPY 1:250, AVP 1:750) or Dylight-594 goat anti-mouse IgG (BDNF 1:11,000; COX-2 1:1000; HSP-70 1:750) (Thermo Fisher Scientific Inc., Waltham, MA, USA) in PBS containing 3% normal goat or mouse serum for 2 h. Sections were washed and mounted with mounting medium (Vectrastain Vector laboratories, Burlingame, CA, USA).

For Iba-1 staining, sliced sections were air dried and were incubated for 120 min in a permeabilization solution: 0.3% TritonX-100 in PBS (PBS/T). After washes in PBS/T, the sections were incubated for 120 min in a blocking solution: 1% BSA and 0.3% PBS/T, and then were incubated overnight at 4 °C with primary antibody against Iba-1 (rabbit polyclonal anti-Iba-1 antiserum (1:10,000), product code: 019-19741, Fujifilm Wako Chemicals Europe GmbH, Neuss, Germany) 1:10,000 in 1% BSA + 0.3% PBS/T. After three washes in PBS/T, sections were incubated in DyLight-488 labeled goat-anti-rabbit IgG (1:10,000; Thermo Fisher Scientific Inc., Waltham, MA, USA) in 0.3% PBS/T Sections were washed and mounted with mounting medium (Vectrastain Vector laboratories, Burlingame, CA, USA). Control staining was performed in the absence of the primary antibodies.

For GR staining: Sliced sections were air dried and incubated in cold methanol (−20 °C) (4 min). Then, they were washed twice in PBS and once in 0.3% PBS/T (Sigma-Aldrich, Rehovot, Israel). The sections were incubated for 120 min in a blocking solution containing 0.3% PBS/T and 2% BSA, and then incubated for 48 h at 4 °C with primary antibody against GR (mouse monoclonal anti-GR antiserum (1:1000), product code: sc-56851, Santa Cruz Biotechnology, Heidelberg, Germany). After three washes in PBS/T, sections were incubated in DyLight-488 labeled goat anti-mouse IgG (1:1000; Thermo Fisher Scientific Inc., Waltham, MA, USA) in PBS containing 2% BSA and 0.3% TX-100 for 2 h. Sections were washed and mounted with mounting medium (Vectrastain Vector laboratories, Burlingame, CA, USA). Control staining was performed in the absence of the primary antibodies. Fluorescent labels were swapped to test cross-reactivity, and sections were incubated without primary antibodies to assess non-specific binding of the secondary antibodies. The sections were analyzed by two observers blinded to the treatment protocol.

### 4.7. Quantification

Each brain region was defined under the microscope according to cytoarchitectural landmarks [97]. Regions of interest were outlined, and computer-aided estimation was used to calculate the number of BDNF-ir, GR-ir, COX-2-ir, HSP-70-ir, Iba-1-ir and AVP-ir positive cells within the pyramidal layer of the CA 1 and CA3 and the granular layer of the DG of the hippocampus, and the hypothalamic PVN. Measurements occurred in a 50,000-mm^2^ area in each area of interest and were digitized using microscopic images (Leica microscope DM4500B; Leica Microsystems, Wetzlar, Germany) and a DFC340FX digital imaging camera (Leica Microsystems, Wetzlar, Germany). Measurements were taken in each sub-region from both brain hemispheres. The number of positive cells expressing COX-2, HSP-70, Iba-1 and BDNF, were determined with the Leica LAS software (version 3.8). Sections from the brains of different groups of rats were processed at the same time and under identical conditions to ensure reliable comparisons and to maintain stringency in tissue preparation and staining conditions. Seven representative sections of the hippocampus were chosen (between Bregma −2.30 and Bregma −3.60) from each animal, from each group.

Relative quantitative analysis of NPY-ir: The density of fibers and cells expressing NPY-ir in each area was determined with the Leica LAS software (version 3.8). To compensate for background staining levels and to control for variations in the overall illumination level between images, the average pixel density of two regions that presumably contained only nonspecific staining (i.e., in areas that are near each area of interest that is not thought to contain NPY) was determined within each captured image, and this value was subtracted from all density measurements performed on that image.

### 4.8. Regions of Interest

The hippocampus and the hypothalamic PVN were chosen as targets for this study. In general, these regions have been previously demonstrated to be particularly vulnerable to the toxic effects of stress [98,99]. In addition, temperature and osmoregulatory centers are located in the hypothalamus [100]. The hypothalamic PVN is an important integrative site regulating the HPA axis [22], and is involved in hormonal, endocrine and neural control [101,102]. The hypothalamic PVN is also a critical central nucleus regulating reflex renal vasoconstriction in response to elevations in core body temperature [101,102].

### 4.9. Genes of Interest

The expression of seven stress- and heat-related genes was assessed within the pyramidal layer of the CA1 and CA3 and the granular layer of the DG in the hippocampus, and the hypothalamic PVN. These included GR, HSP-70 and AVP (mediators of stress and thermoregulation). In addition, BDNF and NPY were examined. These neurotrophins play an important role in neuroplasticity and in the control of temperature regulation. Finally, two markers of inflammation that are linked to thermoregulation (COX-2 and Iba-1) were also measured.

### 4.10. Predator Scent Stress (PSS)

The PSS is a well-established model of psychological stress that is used for the study of post-traumatic stress disorder [94,95]. It consists of placing the test animals on heavily soiled cat litter (used by a cat for 2 days then sifted to remove stools) for 15 min in a closed environment.

### 4.11. Behavioral Assessments

All behavioral tests were performed in a closed, quiet, light-controlled room in the Faculty of Medicine, Anxiety and Stress Research Unit, Ben-Gurion University between 10:00 and 16:00 h, as previously described [103]. All behavioral tests were video-recorded for future analysis using the ETHO-VISION program (Noldus), by an investigator blinded to the experimental protocol. As previously described, behavioral assessment took place immediately after exposure to PSS for an acute response analysis and seven days after the exposure to PSS to examine the long-term response analysis.

Elevated-plus maze: The EPM consists of a plus-shaped platform with two open arms and two closed arms—surrounded by 14-cm high opaque walls on three sides, with arms of the same type located opposite each other. Procedure: Each rat was placed on the central platform facing an open arm and was allowed to explore the maze for 5 min. Each test was videotaped and recorded by an independent observer. Arm-entry was defined as entering the arm with all four paws. The following parameters were measured: duration in open and closed arms, and on the central platform; open and closed arm entries; and total entries into all arms (=total exploration). “Anxiety Index”, an index that integrates the EPM behavioral measures, was calculated as follows:

$$\begin{array}{c} Anxiety\;Index\\ =1-\left[\frac{\left(\frac{time\;spent\;in\;the\;open\;arms}{total\;time\;on\;the\;maze}\right)+\left(\frac{number\;of\;enteries\;to\;the\;open\;arms}{total\;exploration\;on\;the\;maze}\right)}{2}\right] \end{array}$$

Acoustic startle response: Startle response was measured using two ventilated startle chambers (SR-LAB system, San Diego Instruments, San Diego, CA). The SR-LAB calibration unit was used routinely to ensure consistent stabilimeter sensitivity between test chambers and over time. Each Plexiglas cylinder rests on a platform inside a sound-proofed, ventilated chamber. Movement inside the tube is detected by a piezoelectric accelerometer below the frame. Sound levels within each test chamber are measured routinely using a sound level meter (Radio Shack) to ensure consistent presentation. Each test session started with a 5 min acclimatization period to background white noise of 68 dB, followed by 30 acoustic startle trial stimuli in six blocks (110 dB white noise of 40 ms duration with 30 or 45 s inter-trial interval). Behavioral assessment consisted of: mean startle amplitude (average over all 30 trials) and percent of startle habituation to repeated presentation of the acoustic pulse. Percent habituation—the percent change was calculated between the response to the first and last (6th) blocks of sound stimuli, as follows:

$$\begin{array}{c} Percent\;Habituation\\ =100\\ \times \left[\frac{\left(average\;startle\;amplitude\;in\;Block\;1\right)+\left(average\;startle\;amplitude\;in\;Block\;6\right)}{average\;startle\;amplitude\;in\;Block\;1}\right] \end{array}$$

### 4.12. Measurement of Circulating Corticosterone

At completion of experiment 1, rats (*n* = 20) whose brains were not harvested were decapitated with a guillotine. Situational stress was minimized by thoroughly cleaning the area between sacrifices and removing the bodies. Trunk blood was collected, left at room temperature for 1 h, and then centrifuged at 1000× *g* for 15 min at 4 °C. Serum was collected and stored at −80 °C. Corticosterone was measured with a DSL-10-81000 ELISA kit (Diagnostic Systems Laboratories, Webster, TX, USA) according to the manufacturer’s instructions by a person blind to the experimental procedures. All samples were measured in duplicate.

### 4.13. Statistical Analyses 

For behavioral, physiological and cellular measures, statistical analyses were performed using a one-way analysis of variance (ANOVA). Time course of Tre and BM changes was analyzed by repeated measures (RM) ANOVA. Tre and BM changes in response to HS were analyzed as percent change from baseline value. In the event of a significant F ratio, a Bonferroni post-hoc analysis was used for pairwise comparisons. All data are reported as mean ± SEM. An α level of *p* < 0.05 was used to determine statistical significance.

## 5. Conclusions

The current investigation focused on dynamic and central responses to an acute exposure to HS. The main findings of this investigation included: (1) HS resulted in activation of the HPA-axis, both peripherally and centrally, and led to a several fold increase in corticosterone levels. (2) Extensive cellular changes were noted after a single exposure to an acute HS in the hippocampus and hypothalamic PVN. These changes, however, could be interpreted as either an unregulated event to thermal damage to central homeostatic sites, or as an adaptive thermoregulatory mechanism. (3) The thermoregulatory survival mechanism in response to HS was linked to increased levels of central HSP-70. (4) HS-preconditioning may, at least in some cases, provide behavioral resiliency against psychological stress. (5) Prior overexpression of HSP-70 (e.g., via preconditioning) affords protection against tissue damage and thus plays indispensable roles in the protective effects of HS. Therefore, targeting of HSP-70 is an attractive strategy for stress-related psychopathology treatment.

## Data Availability

Not applicable.

## Abbreviations

ANOVA	Analysis of variance
ASR	Acoustic startle response
AVP	Arginine vasopressin
BM	Body mass
BDNF	Brain-derived neurotrophic factor
CNS	Central nervous system
Tb	Core body temperature
CA	Cornu Ammonis
COX	Cyclooxygenase
DG	Dentate gyrus
EPM	Elevated plus-maze
GC	Glucocorticoid
GR	Glucocorticoid receptor
HSP	Heat shock protein
HS	Heat-stress
HPA	Hypothalamus–pituitary–adrenal
Ir	Immunoreactive
Iba-1	Ionized calcium binding adaptor molecule 1
NPY	neuropeptide-Y
PSS	Predator scent stress
PVN	Paraventricular nucleus of the hypothalamus
TN	Thermoneutral naïve
Tre	Rectal temperature
